# Pattern of fall injuries in Pakistan: the Pakistan National Emergency Department Surveillance (Pak-NEDS) study

**DOI:** 10.1186/1471-227X-15-S2-S3

**Published:** 2015-12-11

**Authors:** Jabeen Fayyaz, Shirin Wadhwaniya, Hira Shahzad, Asher Feroze, Nukhba Zia, Mohammed Umer Mir, Uzma Rahim Khan, Sumera Iram, Sabir Ali, Junaid Abdul Razzak, Adnan A Hyder

**Affiliations:** 1 Department of Emergency Medicine, Aga Khan University, Karachi, Pakistan; 2 Johns Hopkins International Injury Research Unit, Department of International Health, Johns Hopkins Bloomberg School of Public Health, Baltimore, MD, USA; 3 Global Health Systems & Development, Tulane University School of Public Health, New Orleans, LA, USA; 4 Department of Emergency Medicine, Mayo Hospital, Lahore, Pakistan; 5 Department of Emergency Medicine, John Hopkins School of Medicine, Baltimore, MD, USA; 6 The author was based in Department of Emergency Medicine, Aga Khan University, Karachi, Pakistan at the time of writing of the manuscript

**Keywords:** Fall injuries, emergency department, surveillance, Pakistan

## Abstract

**Background:**

We aimed to analyse the frequency and patterns of fall-related injuries presenting to the emergency departments (EDs) across Pakistan.

**Methods:**

Pakistan National Emergency Departments surveillance system collected data from November 2010 to March 2011 on a 24/7 basis using a standardized tool in seven major EDs (five public and two private hospitals) in six major cities of Pakistan. For all patients presenting with fall-related injuries, we analysed data by intent with focus on unintentional falls. Simple frequencies were run for basic patient demographics, mechanism of falls, outcomes of fall injuries, mode of arrival to ED, investigations, and procedures with outcomes.

**Results:**

There were 3335 fall-related injuries. In cases where intent was available, two-thirds (n = 1186, 65.3%) of fall injuries were unintentional. Among unintentional fall patients presenting to EDs, the majority (76.9%) were males and between 15-44 years of age (69%). The majority of the unintentional falls (n = 671, 56.6%) were due to slipping, followed by fall from height (n = 338, 28.5%). About two-thirds (n = 675, 66.6%) of fall injuries involved extremities, followed by head/neck (n = 257, 25.4%) and face (n = 99, 9.8%). Most of the patients were discharged from the hospital (n = 1059, 89.3%). There were 17 (1.3%) deaths among unintentional fall cases.

**Conclusion:**

Falls are an important cause of injury-related visits to EDs in Pakistan. Most of the fall injury patients were men and in a productive age group. Fall injuries pose a burden on the healthcare system, especially emergency services, and future studies should therefore focus on safety measures at home and in workplaces to reduce this burden.

## Background

Globally, the number of injury deaths increased by 24% in the last decade with falls claiming about 540,000 global deaths in 2010 [1]. In 1990, falls were the 31st leading cause of global death and in the year 2013, it rose to become the 28th leading cause of global death [1]. Mortality due to falls in the Eastern Mediterranean Region is reported as 2.9 per 100,000 population which is the highest among all World Health Organization regions [2].Taking into consideration the morbidity associated with injuries, falls account for 12.2% of the injury-related disability-adjusted life years (DALYs) and hence cause large financial and productivity deficits [3].

Although fall injuries are commonly reported in older age groups, regional studies have shown that younger populations (15-49 years), have been identified as the most susceptible group affected by fall-related injuries, with males twice as much affected compared to females [4-8]. Fall from height has also been recognized as one of the more common modes of injuries in lower-income nations [4,9-11]. Household surveys of Asian countries showed a high morbidity and disability in children due to falls leading to financial as well as social burdens on the families of patients [12]. A recent study from Malaysia reported reduced functionality and increased mortality one year after a fall injury in elderly patients who presented to EDs after sustaining a fall [13].

Injury-related mortality and morbidity has not gained due attention because the existing data on unintentional injuries often focus on motor vehicle injuries or lack data in the subcategory of fall-related injuries [14]. The National Injury Survey of Pakistan calculated the annual incidence of fall-related injuries as 8.85 per 1000 population per year [11]. Almost 87% of these falls were found to affect the younger population, especially children under 15 years of age, hence signifying a substantial public health and economic challenge [11]. However, little is known about the pattern of fall-related injuries, ED management, and outcomes in patients presenting to EDs in lower-income countries [9,15].

This study analyses the frequency and patterns of fall-related injuries presenting to seven major EDs in Pakistan. This study exclusively emphasizes unintentional fall-related injuries and describes the demographic patterns, injury characteristics, outcomes, and healthcare provision within this patient population. Intentional injuries where a fall was recorded as the mechanism are not included in this study.

## Methods

The Pak-NEDS study was a pilot active surveillance carried out in seven major EDs in six major cities of Pakistan including Karachi, Lahore, Islamabad, Rawalpindi, Peshawar, and Quetta. All these sites are tertiary-care urban centers; five are public hospitals and two are private. Ethical approval was obtained from all participating sites.

Data was collected from November 2010 to March 2011 on all patients who presented to the EDs of the participating sites to seek care. A one-page standardized tool was developed based on an ambulatory care survey tool from the Center for Disease Control and Prevention, USA and from previous surveillance work done in Pakistan [16,17]. This tool had questions related to patient demographics such as age, gender, and ethnicity; presenting complaints; treatment and management provided in the ED; provisional diagnosis; and disposition from the ED. Pak-NEDS had 24/7 coverage and trained data collectors worked in three shifts. Data was collected through an interview with the patient or next of kin and from ED records. Informed verbal consent was taken. Information was collected once the patient had received the initial treatment with no compromise on the management and treatment of patients. Site supervisors and project supervisors performed random checks on 5% of the data to ensure data quality.

Data was entered using EpiInfo version 3.3.2 and analysis was carried out using SPSS version 20 and STATA version 12 [18-20]. Simple frequency was run for patient demographic characteristics and cross-tabulation of intent was performed with mechanism, nature of injury, disposition, and resource utilization. Chi square (χ^2^) tests were used to compare intentional falls and unintentional falls by demographic factors, injury characteristics, and outcomes. All the variables used for determining resource utilization among fall injury patients were multiple response variables. Age was divided into five categories: under 5 years, 5 - 14 years, 15 - 24 years, 25 - 44 years, 45 - 65 years, and more than 65 years. A p-value of <0.05 was considered statistically significant.

## Results

### Intent of fall-injury

There were 3335 patients with fall-related injuries. The information related to intent of injury was available for 54.4% (n = 1815) of these fall injury patients. Of these, two-thirds of the fall injuries were unintentional in nature (n = 1186, 65.3%) and about 34.7% (n = 629) of falls were intentional in nature. Among intentional injuries with falls, 29.7% (n = 187) were self-inflicted; and 70.3% (n = 442) were related to assault. All analyses were conducted by intent of injury. However, this paper and the following results and discussion focus on 'unintentional fall injuries,' henceforth referred to as fall injuries.

### Patient Demographics

**Figure 1:**
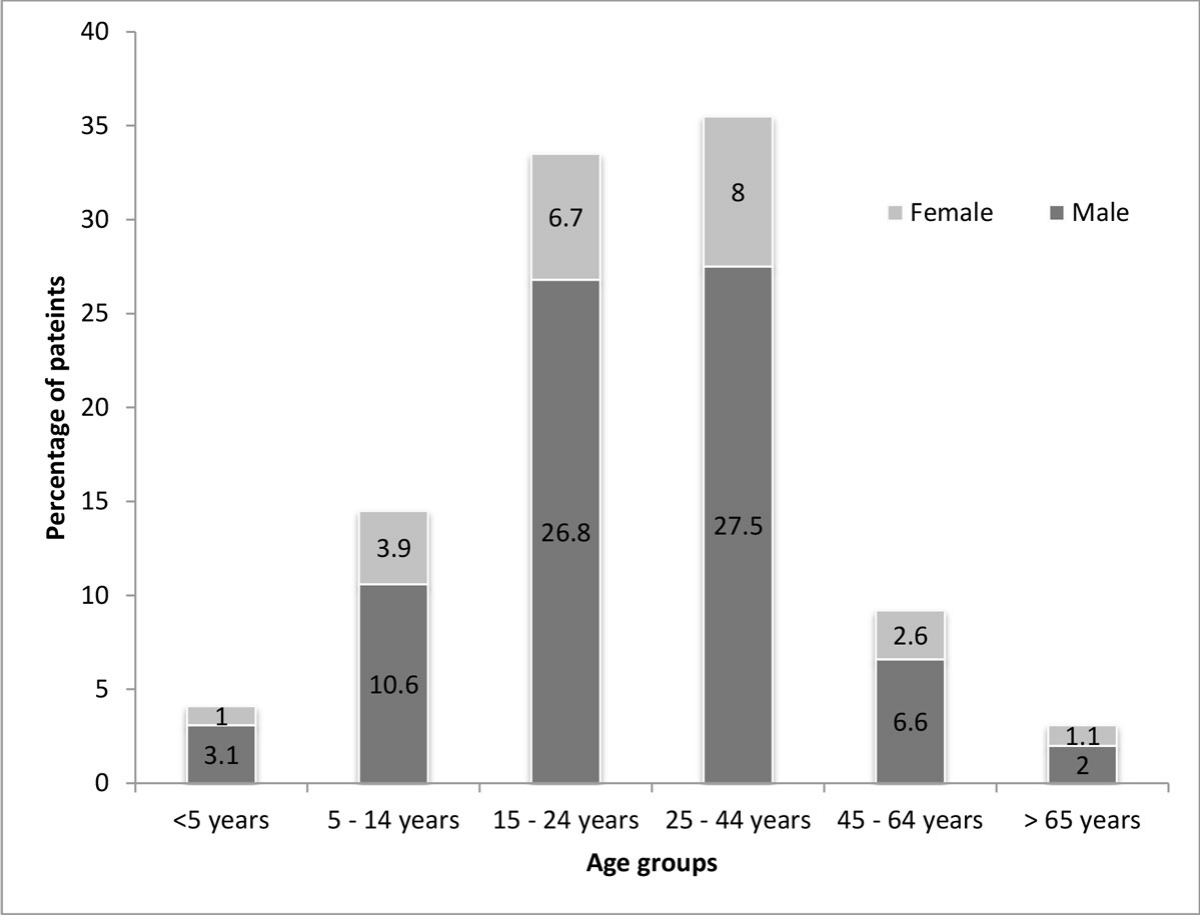
**Age groups and gender stratification (n = 1095)**.

Of the total unintentional falls patients who presented to the ED, 76.9% were males (n = 898), with a male to female ratio of 3.3: 1. Most of these patients (n = 765, 69%) were 15-44 years old and males remained the predominant gender in all age categories. This distribution is comparable to the age distribution of the population that visited the EDs of participating sites. However, compared to the overall ED population, a higher proportion of 5 - 24 years old and a lower proportion of 25 - 64 years old visited EDs for fall injuries (Figure 1). The median age was 25 years (interquartile range 19 - 38 years). Only 7.3% (n = 81) were brought to the EDs via ambulances. Most of these patients (n = 1163, 98.1%) came to a public hospital (Table 1).

**Table 1:** Demographic characteristics of fall injury patients in Pakistan (n = 3335)

Variables	Intentional (n = 629) n (%)	Unintentional falls (n = 1186) n (%)	χ^2 ^p-value	Total* (n = 3335) n (%)
**Gender (n = 3184)**				

Male	473 (76.3)	898 (76.9)	0.778	2166 (68.0)
		
Female	147 (23.7)	270 (23.1)		1018 (32.0)

**Age in years (n = 3103)**				

< 5	18 (2.9)	46 (4.2)	0.004	83 (2.7)

5 - 14	76 (12.4)	162 (14.6)		343 (11.1)
		
15 - 24	174 (28.4)	371 (33.5)		866 (27.9)
		
25 - 44	273 (44.6)	394 (35.5)		1365 (44.0)
		
45 - 64	60 (9.8)	102 (9.2)		368 (11.9)
		
>65	11 (1.8)	34 (3.1)		78 (2.5)

**Mode of arrival (n = 3055)**				

Ambulance	101 (17.1)	81 (7.3)	<0.001	260 (8.5)
		
Non-ambulance mode	491 (82.9)	1034 (92.7)		2795 (91.5)

**Type of hospital (n = 3335)**				

Public hospital	624 (99.2)	1163 (98.1)	0.060	3196 (95.8)
		
Private hospital	5 (0.8)	23 (1.9)		139 (4.2)

* missing: 45.6% (n = 1520) for intent, 7% (n = 232) for age, 4.5% (n = 151) for gender, 8.4% (n = 280) for mode of arrival

### Fall injury characteristics

The mechanism of fall was recorded for all cases of unintentional fall injury: 56.6% (n = 671) of fall injuries were due to slipping; about a quarter (n = 338, 28.5%) were from height; and 15% (n = 177) were from tripping and other reasons. The injured body parts at initial presentation were recorded for 85.4% (n = 1013) of cases with fall injuries: about two-thirds (n = 675, 66.6%) of fall injuries involved upper extremities (n = 300, 29.6%) or lower extremities (n = 375, 37.0%), followed by head/neck (n = 257, 25.4%), and face (n = 99, 9.8%) (Table 2). The nature of injury at initial presentation was recorded for 69.6% (n = 826) of the patients: cuts and wounds occurred in 58.8% (n = 486) of the fall injury patients; sprains, strains and bruises in 29.2% (n = 241); and fractures and dislocations occurred in 12.1% (n = 100) of patients (Table 2).

**Table 2:** Injury characteristics and outcomes of fall patients in Pakistan

Variables	Intentional n (%)	Unintentional falls n (%)	χ^2 ^p-value	Total^#^ n (%)
**Mechanism of fall (n = 3335)**				

Fall from height	169 (26.9)	338 (28.5)	0.606	857 (25.7)
		
Fall from tripping	35 (5.6)	79 (6.7)		190 (5.7)
		
Fall from slipping	367 (58.4)	671 (56.6)		1990 (59.7)
		
Fall others	58 (9.2)	98 (8.3)		298 (8.9)

Body parts injured (n = 2327)*				

Head/neck	118 (25.9)	257 (25.4)	-	542 (23.3)
		
Face	42 (9.2)	99 (9.8)		218 (9.4)
		
Upper limb	148 (32.5)	300 (29.6)		834 (35.8)
		
Lower limb	188 (41.2)	375 (37.0)		770 (33.1)
		
Chest/abdomen	28 (6.1)	34 (3.4)		130 (5.6)
		
Other	1 (0.2)	46 (4.5)		47 (2.0)

Nature of injury (n = 1519)*				

Sprain/strains/bruises	61 (13.7)	241 (29.2)	-	405 (26.7)
		
Cuts/wounds	261 (58.8)	486 (58.8)		791 (52.1)
		
Fractures/dislocation	125 (28.2)	100 (12.1)		330 (21.7)
		
Concussion/contusion/Intracranial bleeding	50 (11.3)	65 (7.9)		122 (8.0)
		
Other injuries	6 (1.4)	16 (1.9)		1 (0.1)

**Disposition (n = 2572)**				

Discharged from ED	378 (74.9)	893 (84.3)	0.000	2031 (79.0)
		
Admitted	103 (20.4)	100 (9.4)		415 (16.1)
		
Death	8 (1.6)	17 (1.6)		32 (1.2)
		
Others (Left against medical advice/Left without being seen/Referred out)	16 (3.2)	49 (4.6)		94 (3.7)

*Total >100% as multiple responses were possible

^#^missing: intent for 45.6% (n = 1520), disposition for 22.9% (n = 763)

### Disposition and outcome

Out of a total of 89.3% (n = 1059) fall injury patients whose discharge disposition was available, 1.6% (n = 17) of falls resulted in death, of which 15 were males. Of the remaining, 84.3% (n = 893) of patients were discharged from the ED, 9.4% (n = 100) were admitted, and 4.6% (n = 49) patients either left against medical advice or were referred out (Table 2).

### Healthcare provision and Resource utilization

Data regarding healthcare provision was available for 91.5% (n = 1085) of fall injury patients (Table 3). Almost half of them received paramedic care (n = 570, 52.5%). Medical officers tended to 79% (n = 857) of the patients, and 63.3% (n = 687) received care from nurses and midwives; attending physicians provided care to <2% (n = 16) of patients (Table 3). Records regarding radiographic evaluation in the ED setting were available for 37.5% (n = 445) of the patients: plain X-ray films were obtained in 92.8% (n = 413) of patients, whereas computed tomographic (CT) images were attained in only 13.7% (n = 61) of the patients (Table 3).

**Table 3:** Resource utilization by fall in emergency department in Pakistan

Variables	Intentional n (%)	Unintentional falls n (%)	Total^#^ n (%)
Type of care provider (n = 2996)*			

Paramedic	304 (51.4)	570 (52.5)	1512 (50.5)

House officer/intern	336 (56.8)	302 (27.8)	1092 (36.4)

Post Graduate trainee/Resident	103 (17.4)	101 (9.3)	375 (12.5)

Medical Officer	327 (55.3)	857 (79.0)	1881 (62.8)

Nurse/midwife	400 (67.7)	687 (63.3)	2080 (69.4)

Attending physician	5 (0.8)	16 (1.5)	143 (4.8)

Radiography (n = 1521)*			

X-rays	317 (92.7)	413 (92.8)	1429 (93.9)

Ultrasound	8 (2.5)	5 (1.1)	42 (2.8)

Computed Tomogram	32 (9.4)	61 (13.7)	106 (7.0)

Treatment provided (n = 3335)*			

None	32 (5.1)	76 (6.4)	315 (9.4)

Intravenous fluids	143 (22.7)	244 (20.6)	564 (16.9)

Tetanus toxoid	204 (32.4)	575 (48.5)	909 (27.3)

Analgesia	441 (70.1)	726 (61.2)	2284 (68.5)

Antibiotics	282 (44.8)	354 (29.8)	1075 (32.2)

Cardiopulmonary resuscitation	81 (12.9)	177 (14.9)	285 (8.5)

Plaster/cast	68 (10.8)	91 (7.7)	256 (7.7)

Dressing/suturing/staples/incision and drainage	314 (49.9)	677 (57.1)	1266 (38.0)

Others	23 (3.7)	106 (8.9)	206 (6.2)

*Total >100% as multiple responses were possible

^#^missing: intent for 45.6% (n = 1520), provider for 10.2% (n = 339)

Abbreviations: Activated prothrombin time (APTT); Liver function tests (LFTs); Renal function tests (RFTs); Prothrombin time (PT)

Out of all fall injury patients, 93.6% (n = 1110) received some treatment in the ED while no treatment was required in 6.4% (n = 76) of cases. A large proportion of the patients were provided with some form of analgesia (n = 726, 61.2%) and 29.8% (n = 354) were given antibiotics. Nearly half of the patients (n = 677, 57.1%) required wound care including dressing, suturing or staples, and incision and drainage; 48.5% (n = 575) were given tetanus toxoid (Table 3).

## Discussion

Our study is among few studies in Pakistan describing the burden of fall injuries. This study reports injury characteristics, outcomes, and health service utilization among ED attendees with a presenting complaint of fall. Upon the basis of intent, two-thirds of the falls were unintentional. Although the focus of this paper is on unintentional falls, it is important to know that intentional falls are an important finding especially in the setup of the emergency department. However, intentional fall injuries are generally not recognized in ED setting and thus require an in-depth focus. ED surveillance studies like Pak-NEDS can have the potential to identify such cases [21]. The majority of the fall injury patients were men and in a younger age group. Most falls had resulted from slipping, causing injuries to the extremities. More than three-quarters of the patients were discharged from the ED, but over 1% of the falls resulted in death.

In our study, compared to women, thrice as many men reported to the ED for unintentional fall injuries. Male predominance has been observed in previous studies looking at all injuries as well as falls [3,4,11,22]. This could be because men tend to indulge in risk-taking behaviors more frequently than women [23]. This can also be explained by the fact that in traditional Pakistani households, men play a more active role both inside and outside the house and are predisposed to injury and trauma within the external environment [24,25].

In our study, we found that the age group most commonly affected by fall-related injuries was adolescents and younger adults aged 15-44 years of age. A study from Kathmandu also found the age group of 15-49 years of age to be the most frequently at-risk group for fall-related injuries [4]. Given that this is the most productive age group, fall-related injuries can be a significant socioeconomic challenge for the society. In comparison, extensive literature elaborates on the extremes of age being more commonly injured by falls [26,27]. Older age is recognized to be a risk factor of fall-related injuries and this risk is observed to increase with age [28]. However, in our study, a very small proportion of fall-related injuries were observed in those over the age of 65 years. It is also important to note that the age-distribution of fall patients observed in our study is similar to the age-distribution of the overall patients that attended EDs of the participating sites.

Children and adolescents are also vulnerable to injuries including falls. In Pakistan, injury-related mortality in children and adolescents has been reported to be as high as 30 per 100,000 population, highlighting the significant burden [14]. Among all injuries, the National Health Survey of Pakistan (NHSP 1990-94) found fall-related injuries as the most common mechanism of unintentional injuries among children under five years of age (60%) [29]. A community-based survey from Pakistan carried out in suburban and rural communities also showed that fall injuries were the predominant cause of injuries in all age groups with a prevalence of 10.5 fall injuries per 100 child years of exposure [30]. A multicentre study done in five countries including Pakistan showed that non-fatal falls is the most common mechanism of unintentional injuries in children between 0 to under 11 years of age presenting to the emergency departments of tertiary care hospitals [31,32]. In our study, relatively fewer children reported to the ED for fall injuries. This could be because children with fall injuries may be seeking health services at paediatric hospitals rather than at the EDs of tertiary care centers involved in this study. Also, among all patients that presented to the participating EDs, children constituted a very small proportion, further supporting the possibility that they may be seeking medical services elsewhere. However, further population-based research can help estimate the burden and distribution of fall injuries in different age groups in Pakistan.

Our study showed that limbs (upper and lower) were the most commonly injured body part. These findings are similar to that of a study from rural India [33]. A review of the literature shows that the parts of the body injured during a fall depends on the age of the victim: children less than five years more commonly suffer head injuries; adolescents and younger adults suffer injuries to the limbs (upper more frequently than lower); and adults suffer injuries to the torso [22]. Almost half of the patients were treated and discharged from the ED.

In our study fewer cases of fall mortality were recorded. However, fall injury patients required investigations and most received some treatment. There could be direct healthcare cost associated with fall injuries, and loss of wages and decreased productivity either due to temporary or permanent disability are some examples of indirect costs. A community-based study in Sudan also found fall injuries to have a relatively longer length of hospital stay and number of disability days, both adding socioeconomic burden [34]. Thus, fall injuries can have an effect not just on the individual and the family but also on the society at large. Identification of affected groups, causes, and circumstances or events causing fall injuries can help in planning prevention efforts.

### Limitations and implications for future studies

This is a hospital ED-based study and therefore, we cannot reflect the actual magnitude of fall injuries within the wider community. Since minor injuries may be managed at home or by general practitioners, these mild severity falls may have been under-reported in the tertiary care hospitals included in this study. Secondly, despite all efforts to obtain information, some data were missing, which may have led to underestimation of some aspects of our results. For instance, information on intent was unavailable in 46% of cases. Because data was collected in EDs, some information such as disposition may not have been available at the time of data collection. Also, some patients could have been lost to follow-up due to transfer or discharge, or there may have been human error in collecting data. In future surveillance efforts this can be addressed by recruiting additional personnel to follow up with patients during their hospital stay or collecting contact details of consenting patients to follow up after their discharge. However, all these are resource-intensive strategies. Refresher trainings for data collectors, frequently checking data for consistency and completeness, and providing feedback could also help improve data quality and completeness.

In this study, although data was collected round-the-clock and across multiple sites, it was implemented over only a five-month period and any seasonal variations in the distribution of burden of injuries including fall injuries could not be studied. Future surveillance could be implemented over a period of one year to capture any seasonal variations in the distribution of injuries.

In this surveillance, data were not collected on the location of the fall (at home, school, work place, on the road), activity at the time of fall, circumstances or events leading to the fall, height and surface from where fall occurred, and severity of fall injury. This can help plan prevention efforts. Moreover, although extremes of ages have been previously reported to be more vulnerable to falls, this was not captured in our dataset. Unintentional falls highlight the need for safety guidelines at home, during the commute, and at the workplace. Distraction is a major contributor towards inattention and hence leading to a greater likelihood of being injured unintentionally; among injuries caused from cell phone-related distraction, 72% were fall injuries [35]. In order to avoid these issues, future studies can be directed towards obtaining community-level data with household or community-level surveys that would include mild injuries as well as major ones. Also, specific age groups could be targeted to determine the incidence of fall-related injuries in the population. Moreover, cost of treatment is an area that should be targeted by future studies given that Pakistan is a developing country and most healthcare financial burden is the patient's responsibility.

## Conclusion

Most of the unintentional fall injury patients visiting EDs were men and in the most productive age group (15 - 44 years). Among all injury patients presenting to EDs, fall injuries were relatively non-fatal compared to road traffic injuries, burns, and drowning. Fall injury poses a burden on the healthcare system, especially on emergency services, in developing countries like Pakistan in terms of resources and cost of care.

## Competing interests

The authors declare that they have no competing interests.

## Authors' contributions

JF, HS and NZ developed the initial draft. Data management and analysis was done by AF. SW, UM, URK and SI critically reviewed the draft. SA was a local collaborator for Pak-NEDS and provided critical review of the draft. JAR and AAH was involved in the conception and design of the study, data analysis and interpretation and critical review of this paper. The final draft was approved by all authors
